# Synthesis of Telmisartan Organotin(IV) Complexes and their use as Carbon Dioxide Capture Media

**DOI:** 10.3390/molecules24081631

**Published:** 2019-04-25

**Authors:** Angham G. Hadi, Khudheyer Jawad, Emad Yousif, Gamal A. El-Hiti, Mohammad Hayal Alotaibi, Dina S. Ahmed

**Affiliations:** 1 Department of Chemistry, College of Science, Babylon University, Babil 51002, Iraq; analhusainy@gmail.com (A.G.H.); khudheyer1965@gmail.com (K.J.); 2 Department of Chemistry, College of Science, Al-Nahrain University, Baghdad 64021, Iraq; 3 Department of Optometry, College of Applied Medical Sciences, King Saud University, P.O. Box 10219, Riyadh 11433, Saudi Arabia; 4 National Center for Petrochemicals Technology, King Abdulaziz City for Science and Technology, P.O. Box 6086, Riyadh 11442, Saudi Arabia; mhhalotaibi@kacst.edu.sa; 5 Department of Medical Instrumentation Engineering, Al-Mansour University College, Baghdad 64021, Iraq; dinasaadi86@gmail.com

**Keywords:** adsorption, carbon dioxide capture, gas storage, organotin(IV) complexes, surface area, synthesis

## Abstract

Novel, porous, highly aromatic organotin(IV) frameworks were successfully synthesized by the condensation of telmisartan and an appropriate tin(IV) chloride. The structures of the synthesized organotin(IV) complexes were elucidated by elemental analysis, ^1^H-, ^13^C-, and ^119^Sn-NMR, and FTIR spectroscopy. The surface morphologies of the complexes were inspected by field emission scanning electron microscopy. The synthesized mesoporous organotin(IV) complexes have a Brunauer–Emmett–Teller (BET) surface area of 32.3–130.4 m^2^·g^−1^, pore volume of 0.046–0.162 cm^3^·g^−1^, and pore size of around 2.4 nm. The tin complexes containing a butyl substituent were more efficient as carbon dioxide storage media than the complexes containing a phenyl substituent. The dibutyltin(IV) complex had the highest BET surface area (S_BET_ = 130.357 m^2^·g^−1^), the largest volume (0.162 cm^3^·g^−1^), and was the most efficient for carbon dioxide storage (7.1 wt%) at a controlled temperature (323 K) and pressure (50 bars).

## 1. Introduction

The emission of greenhouse gases, for example, carbon dioxide (CO_2_), from the increased use of fossil fuels is responsible for the increasing temperatures on the earth. A high level of CO_2_ in the atmosphere leads to global warming [1,2,3], which causes a disturbance in nature’s equilibrium. High CO_2_ levels are responsible for drastic climate changes such as melting of the ice at both the north and south poles, an increase in sea level, floods, droughts, and drastic changes in the weather. In addition, a high level of CO_2_ in natural gas can lead to a significant reduction of the natural gas capacity and quality [4], and can damage gas pipes through corrosion [4]. Therefore, CO_2_ capture is vital for both the environment and industry, and has attracted the attention of researchers in both industry and academia [5]. The scientific community is under pressure to find alternative renewable sources of energy which do not contribute to increased levels of CO_2_ in the environment, or to develop new processes and materials that can reduce such harmful emissions from burning fossil fuels.

Some progress has been made in the development of new materials for the capture of greenhouse gases such as CO_2_ [6,7,8,9,10,11,12,13,14,15,16]. Recent technology has concentrated on the selective removal of CO_2_ from natural gas through absorption by chemicals [17]. For example, amines such as ammonia and ethanolamine can be used to absorb CO_2_ from natural gas [18,19]. However, amines are hazardous and have high volatility. Unlike amines, ionic liquids can be used to absorb CO_2_ at high temperatures [20]; however, such an approach suffers from high energy cost for the regeneration of the ionic liquids for reuse, corrosion of containers, and the use of a large volume of water [17]. A CO_2_ capture approach that involves the use of membrane separation using porous materials such as polymers, metal-organic frameworks, mixed matrixes, and inorganics has been developed [21,22]. Such materials are chemically stable, have polar surfaces, large pore sizes, and large surface areas [23,24,25,26]. In addition, the adsorption process is effective, simple, and environmentally friendly. However, it is very challenging and complex because it requires high pressure, multiple stages, and extensive recycling steps. Therefore, efforts have been made to develop novel adsorbents to selectively capture CO_2_ from natural gas. Many chemically stable porous organic polymers have been synthesized using simple procedures and used for the capture of CO_2_ [6,27]. The chemical-looping combustion technique can be used to selectively remove both CO_2_ and H_2_O from the gas stream in the presence of an oxygen carrier (e.g., metal oxides) [28]. Such a process is energy cost-effective, but requires a high pressure to operate. CO_2_ in air could also be removed through a direct air capture technique [29]. This process involves the use of a similar concept to that used in the adsorption technique. Various resins, amine-metal oxides, metal-supported carbonates, and aqueous hydroxides have been tested for the direct capture of CO_2_ [30]. Clearly, progress has been made in the selective removal of CO_2_ from natural gas, but there is still room for further improvement.

Recently, we have shown that polyphosphates can act as an efficient CO_2_ capture media [31]. In addition, we have investigated the synthesis and successful use of several organotin(IV) complexes as efficient photostabilizers for polymeric films [32,33] as part of our research on polymers [34,35,36,37,38,39,40,41]. In the current work, we report the synthesis of several mesoporous organotin(IV) complexes that contain both aliphatic (butyl) and aromatic (phenyl) substituents using simple and efficient procedures, and their successful use as CO_2_ capture media at 323 K and 50 bars. In addition, telmisartan is highly aromatic, containing both heterocycles and aryl rings and different aliphatic substituents (methyl and propyl groups) which are essential to increase the surface area and storage capacity. To the best of our knowledge, this is the first report for the use of organotin(IV) complexes as carbon dioxide capture media which turned to be efficient as for metal-organic frameworks.

## 2. Results and Discussion

### 2.1. Synthesis of Organotin(IV) Complexes ***1**–**4***

**Figure 1:**
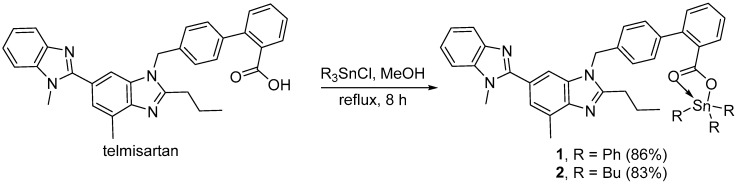
Synthesis of triorganotin(IV) complexes **1** and **2**.

**Figure 2:**
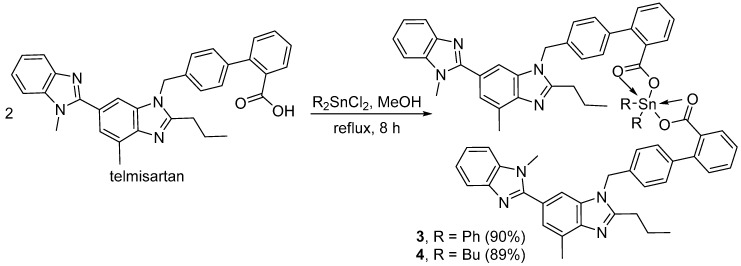
Synthesis of diorganotin(IV) complexes **3** and **4**.

Four organotin(IV) complexes, **1**–**4**, were synthesized from the reaction of telmisartan and the appropriate tin(IV) chloride salts. The reaction of an equimolar mixture of telmisartan and triphenyl(IV) chloride or tributyltin(IV) chloride in methanol under reflux for 8 h gave the corresponding (telmisartan)triorganotin(IV) complex **1** or **2** in 86% and 83% yield, respectively (Figure 1). Similarly, the reaction of telmisartan (two mole equivalents) and diphenyltin(IV) chloride or dibutyltin(IV) chloride in methanol under reflux for 8 h gave the corresponding *bis*(telmisartan)diorganotin(IV) complex **3** or **4** in 90% and 89% yield, respectively (Figure 2). Some of the physical data for organotin(IV) complexes **1**–**4,** along with their elemental analyses, are shown in Table 1.

Table 1 shows that the melting point for triorganotin(IV) complex **2** was noticeably higher than that for complex **2**. The variation in melting could be due the high stability of complex **2** compared with that for complex **1,** since it contains the flexible butyl groups compared with the bulky phenyl groups in complex **1**. In contrast, diorganotin(IV) complex **3,** which contains two phenyl groups, has a higher melting point compared with that for complex **4**, which contains two butyl groups. For the diorganotin(IV) complexes **3** and **4**, only two substituents present and the steric hindrance becomes less important compared with that for triorganotin(IV) complexes **1** and **2**.

### 2.2. FTIR Spectroscopy of Organotin(IV) Complexes ***1**–**4***

The FTIR spectra of complexes **1**–**4** show characteristic peaks within the 526–536 and 445–447 cm^−1^ region that correspond to the vibrations of Sn–C and Sn–O groups, respectively [42]. They also show strong absorption peaks (1685–1697 cm^−1^) corresponding to the vibrations of the carbonyl groups. The key FTIR spectral data of complexes **1**–**4** are shown in Table 2 (see Supplementary Materials for details).

### 2.3. NMR Spectroscopy of Organotin(IV) Complexes ***1**–**4***

The structures of organotin(IV) complexes **1**–**4** were confirmed by NMR spectroscopy (see Supplementary Materials for details). The NMR spectra show all the expected signals at the expected chemical shifts (Table 3). However, the ^13^C-NMR spectra of **1**–**4** show the overlap of various signals within the aromatic region (Table 4). The ^119^Sn-NMR spectra of **1**–**4** show the presence of singlet signals at the –185.0 to –276.0 ppm region (Table 3), which is significantly lower than that for the corresponding organotin(IV) salts. However, the chemical shift is dependent on the geometry of the complex [43,44], and these chemical shifts are consistent with the hypothesis of an increase in the tin atom coordination number within the complexes (i.e., tin nuclear shielding) [45].

### 2.4. Field Emission Scanning Electron Microscopy (FESEM) of Organotin(IV) Complexes ***1**–**4***

**Figure 3:**
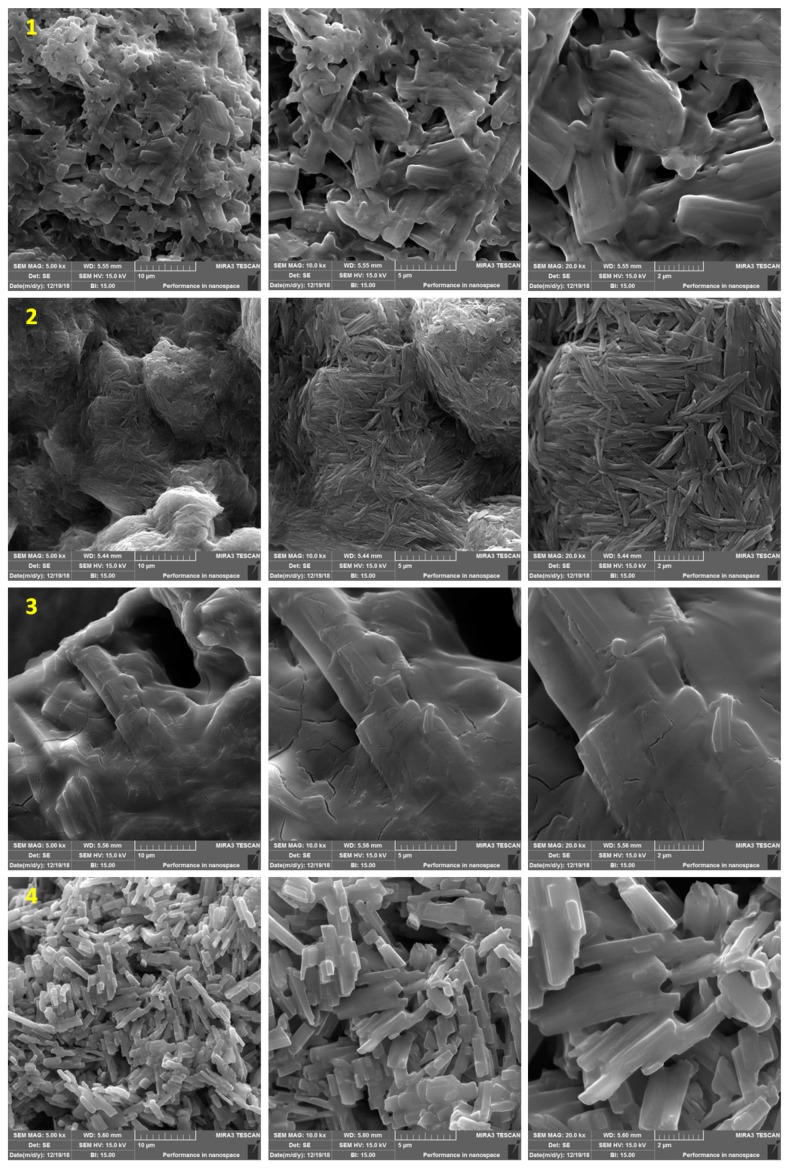
Field emission scanning electron microscopy (FESEM) images of **1**–**4**.

The morphology of the synthesized organotin(IV) complexes **1**–**4** was inspected by FESEM. The images (Figure 3) reveal that complexes **1**–**4** have homogeneous and porous structures. In addition, the images show the presence of tiny particle agglomerates, and the organotin(IV) complexes have different shapes and particles sizes. The particle sizes were calculated to be 24.56–34.13, 28.66–49.66, 23.50–32.94, and 19.68–51.47 nm for complexes **1**, **2**, **3**, and **4**, respectively. Organotin(IV) complex **3** has a lower porosity and more surface smoothness than the other organotin(IV) complexes.

### 2.5. Pure Gas Adsorption of Organotin(IV) Complexes ***1**–**4***

**Figure 4:**
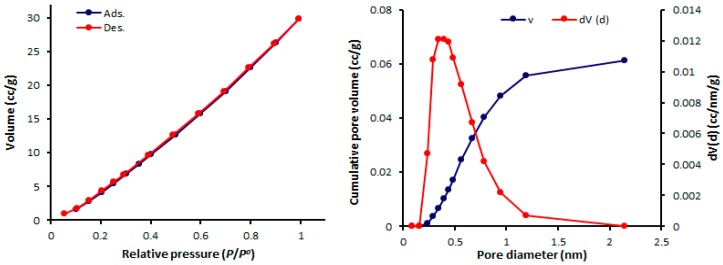
N_2_ isotherms and pore diameters of **1**.

**Figure 5:**
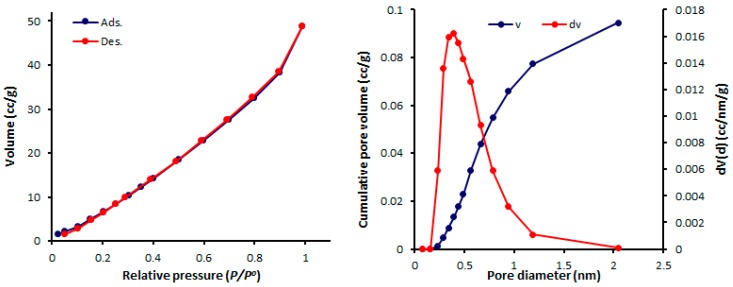
N_2_ isotherms and pore diameters of **2**.

**Figure 6:**
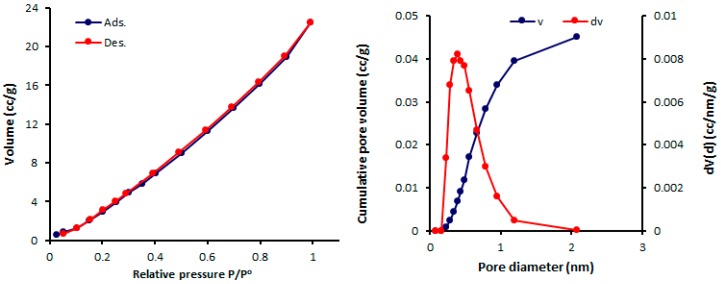
N_2_ isotherms and pore diameters of **3**.

**Figure 7:**
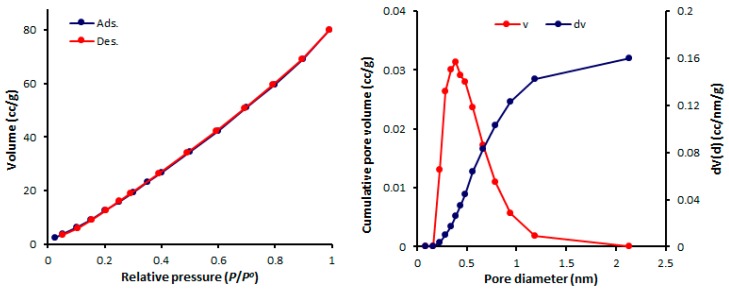
N_2_ isotherms and pore diameters of **4**.

The physisorption isotherms of a gas can be determined either from the amount of gas removed in the gas phase or directly from the amount of gas uptake. The latter process is based on the gravimetric measurement of the adsorbent mass change [46]. The pore textural properties of complexes **1**–**4** were measured from the N_2_ adsorption–desorption graphs recorded at 77 K. In addition, the pore size of mesoporous complexes **1**–**4** were calculated from the adsorption–desorption isotherms using the Barrett–Joyner–Halenda (BJH) method. Organotin(IV) complex networks **1**–**4** show the formation of mesoporous structures and type III nitrogen sorption isotherms. Such isotherms have no identifiable monolayer formation [31]. The N_2_ isotherms and pore sizes of complexes **1–4** are shown in Figure 4, Figure 5, Figure 6 and Figure 7.

The mesopore size distribution can be calculated either from the desorption or adsorption branch of the isotherm. Organotin(IV) complexes **1**–**4** have small mesopores with consistent pore sizes (2.428–2.433 nm). Organotin(IV) complex **4** has the highest Brunauer–Emmett–Teller surface area (S_BET_ = 130.357 m^2^·g^−1^) and the largest volume (0.162 cm^3^·g^−1^) of the organotin(IV) complexes. The porosity properties of complexes **1**–**4** are listed in Table 5.

**Figure 8:**
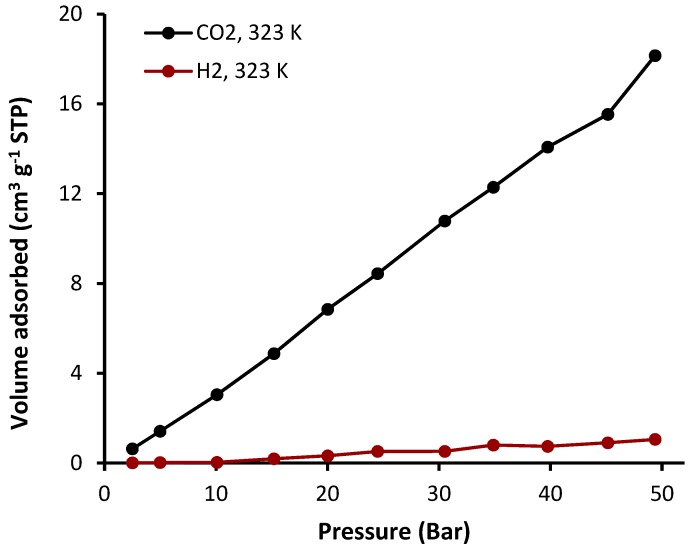
Adsorption isotherms of CO_2_ and H_2_ for complex **1**.

**Figure 9:**
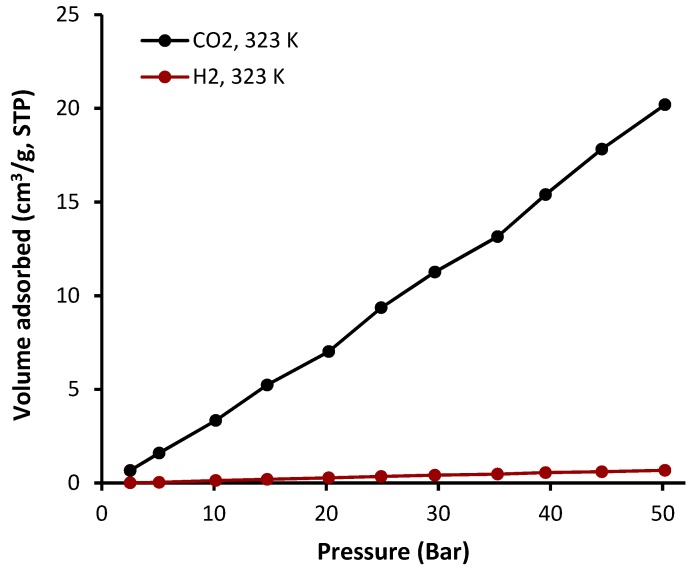
Adsorption isotherms of CO_2_ and H_2_ for complex **2**.

**Figure 10:**
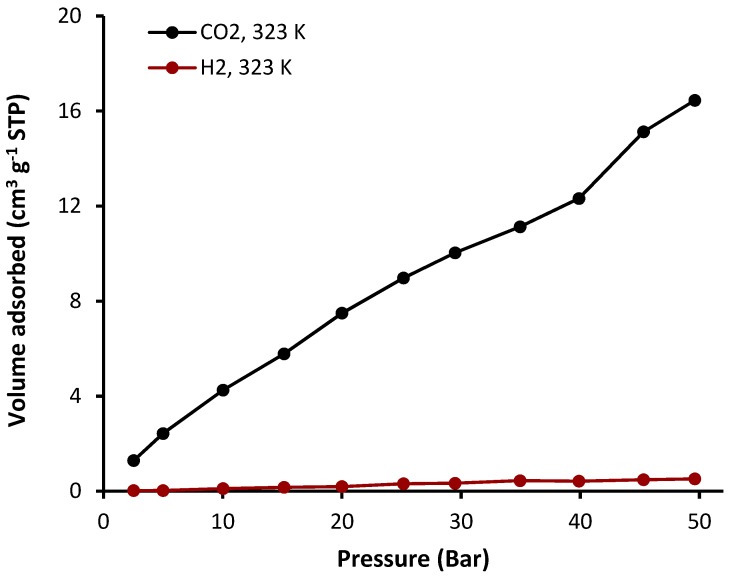
Adsorption isotherms of CO_2_ and H_2_ for complex **3**.

**Figure 11:**
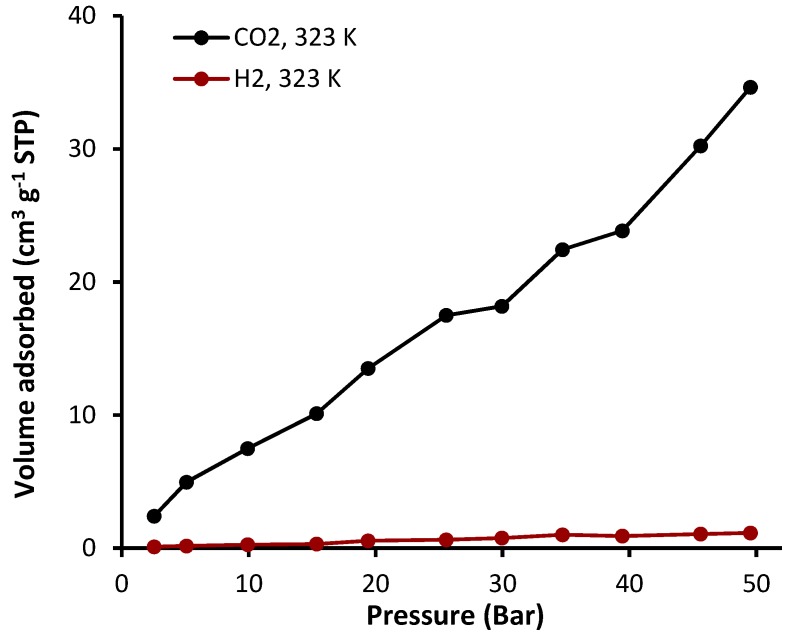
Adsorption isotherms of CO_2_ and H_2_ for complex **4**.

The sorption of complexes **1**–**4** was investigated at a constant temperature (323 K) and pressure (50 bars). The sorption isotherms of CO_2_ and H_2_ in the presence of complexes **1**–**4** are shown in Figure 8, Figure 9, Figure 10 and Figure 11 and the gas uptakes are listed in Table 6. Complexes **1**–**4** showed a high CO_2_ uptake, possibly as a result of the strong van der Waals interaction between such complexes and CO_2_. The quantity of adsorbed CO_2_ was 18.2, 20.5, 16.5, and 35.0 cm^3^·g^−1^ for complexes **1**, **2**, **3**, and **4**, respectively. Clearly, complex **4** has the highest CO_2_ uptake capacity (7.1 wt%) of the organotin(IV) complexes, possibility because it has the largest BET surface area (S_BET_ = 130.357 m^2^·g^−1^). In addition, strong hydrogen bonding and/or dipole-quadrupole interactions between CO_2_ and heteroatoms within the organotin(IV) complexes could take place [47]. Indeed, porous organic polymers containing oxygen, nitrogen, or sulfur atoms are efficient for the selective capture of CO_2_ over other gases such methane and nitrogen [48,49,50]. However, complexes **1**–**4** show very low adsorption for H_2_ (0.5–1.1 cm^3^·g^−1^) under identical conditions to those used for the CO_2_ uptake. Such behavior could be due to the weak interaction between the complexes and H_2_.

Complexes **1**–**4** have different geometries and substitution groups (phenyl and butyl), and therefore showed varied gas capture efficiency. It is clear that the butyl-substituted complexes (**2** and **4**) have better storage capacities than the phenyl-substituted complexes (**1** and **3**). The butyl group is flexible and leads to a larger surface area in complexes **2** and **4** (S_BET_ = 68.357–130.357 m^2^·g^−1^) compared with that of phenyl-substituted complexes **1** and **3** (S_BET_ = 32.374–46.338 m^2^·g^−1^). In complex **4**, the flexible substitution unit (butyl group) points into the channels and leads to the formation of a molecular gate for gas adsorption [51]. It has been reported that the inclusion of alkyl chain substituents within the metal-organic frameworks can be used to tune the pores size to allow a better accommodation of gas molecules within the pores [12,52].

## 3. Materials and Methods

### 3.1. General

Chemicals were purchased from Merck (Schnelldorf, Germany). FTIR spectra (400–4000 cm^−1^) were recorded on an FTIR 8300 Shimadzu spectrophotometer (Tokyo, Japan) using KBr discs. An EM-017mth instrument was used to perform the elemental analyses. An MPD Mitamura Riken Kogyo apparatus (Tokushima, Japan) was used to determine melting points. ^1^H-(300 MHz), ^13^C-(75 MHz), and ^119^Sn-(107 MHz) NMR spectra were recorded on a Bruker DRX300 NMR spectrometer (Zurich, Switzerland). The FESEM images were recorded on a TESCAN MIRA3 FESEM system (Kohoutovice, Czech Republic) at an accelerating voltage of 26 kV. A Quantchrome chemisorption analyzer was used to record the nitrogen adsorption–desorption isotherms (77 K). The samples were dried at a vacuum oven 70 °C for 6 h under a flow of nitrogen. The pore volumes were determined at a relative pressure (*P/P°*) of 0.98. The BJH method was used to verify the pore size distributions. Gas uptakes were performed on an H-sorb 2600 high pressure volumetric adsorption analyzer (Beijing, China) at 50 bars and 323 K.

### 3.2. Synthesis of Triorganotin(IV) Complexes ***1*** and ***2***

A solution of telmisartan (0.51 g, 1.0 mmol) in chloroform (20 mL) was added slowly to a stirred solution of triphenyl or tributyltin chloride (1.0 mmol) in ethanol (10 mL) and the mixture was refluxed for 8 h. The solid obtained upon cooling was collected and recrystallized from methanol to give triorganotin(IV) **1** or **2**.

### 3.3. Synthesis of Diorganotin(IV) Complexes ***3*** and ***4***

A solution of telmisartan (1.03 g, 2.0 mmol) in chloroform (30 mL) was added slowly to a stirred solution of diphenyl or dibutyltin chloride (1.0 mmol) and the mixture was refluxed for 8 h. The solid obtained upon cooling was collected and recrystallized from methanol to give diorganotin(IV) **3** or **4**.

## 4. Conclusions

Four new organotin(IV) complexes containing telmisartan were synthesized and their structures were confirmed. The organotin(IV) complexes have predominantly mesoporous and heteroatom-rich structures. The performance and affinity of the tin complexes for carbon dioxide gas uptake were highly efficient compared with the performance and affinity for hydrogen gas uptake under the conditions used. The dibutyl organotin(IV) complex was the most efficient carbon dioxide storage medium, having a CO_2_ gas uptake up to 7.1 wt% at 323 K and 50 bar. However, the toxicity and degradability of such a complex, as well as the environmental accumulation of telmisartan, should be tested.

## Figures and Tables

**Table 1:** Physical properties and elemental analysis of **1**–**4**.

Sn(IV) Complex	R	Color	Yield (%)	Melting Point (°C)	Calcd. (Found; %)		
					C	H	N
**1**	Ph	pale yellow	86	163–165	70.93 (71.12)	5.14 (5.13)	6.49 (6.36)
**2**	Bu	white	83	243–245	67.25 (67.46)	7.02 (7.11)	6.97 (7.06)
**3**	Ph	off white	90	237–239	72.06 (71.97)	5.27 (5.36)	8.62 (8.63)
**4**	Bu	off white	89	184–186	70.53 (70.41)	6.08 (5.98)	8.89 (9.00)

**Table 2:** Key FTIR spectral data of complexes **1–4**.

Sn(IV) Complex	FTIR (ν, cm^−1^)				
	C=O	C=N	C=C	Sn–C	Sn–O
**1**	1685	1541	1455	526	447
**2**	1697	1540	1458	528	447
**3**	1697	1543	1456	536	445
**4**	1697	1536	1454	536	447

**Table 3:** ^1^H- and ^119^Sn-NMR spectral data (ppm, DMSO-*d_6_*) of complexes **1**–**4**.

Sn(IV) Complex	^1^H-NMR	^119^Sn-NMR
**1**	1.00 (t, *J* = 7.6 Hz, 3H, Me), 1.83 (quintet, *J* = 7.6 Hz, 2H, CH_2_), 2.61 (s, 3H, Me), 3.17 (m, 2H, CH_2_), 3.81 (s, 3H, Me), 5.62 (s, 2H, CH_2_), 7.26–7.86 (m, 29H, Ar)	–193.0
**2**	0.90 (t, *J* = 7.5 Hz, 9H, 3 Me), 0.99 (t, *J* = 7.7 Hz, 3H, Me), 1.23 (m, 6H, 3 CH_2_), 1.46 (m, 6H, 3 CH_2_), 1.58 (m, 6H, 3 CH_2_), 1.86 (quintet, *J* = 7.7 Hz, 2H, CH_2_), 2.63 (s, 3H, Me), 2.92 (t, *J* = 7.7 Hz, 2H, CH_2_), 3.39 (s, 3H, Me), 5.63 (s, 2H, CH_2_), 7.26–7.74 (m, 14H, Ar)	–185.0
**3**	1.02 (t, *J* = 7.5 Hz, 6H, 2 Me), 1.83 (quintet, *J* = 7.5 Hz, 4H, 2 CH_2_), 2.63 (s, 6H, 2 Me), 2.93 (t, *J* = 7.5 Hz, 4H, 2 CH_2_), 3.40 (s, 6H, 2 Me), 5.63 (s, 4H, 2 CH_2_), 7.28–7.73 (m, 38H, Ar)	–267.0
**4**	0.94–1.01 (m, 12H, 4 Me), 1.21–1.31 (m, 8H, 4 CH_2_), 1.79–1.83 (m, 8H, 4 CH_2_), 2.63 (s, 6H, 2 Me), 2.91 (t, *J* = 7.7 Hz, 4H, 2 CH_2_), 3.80 (s, 6H, 2 Me), 5.61 (s, 4H, 2 CH_2_), 7.21–7.86 (m, 28H, Ar)	–242.5

**Table 4:** ^13^C-NMR Spectral data (ppm, DMSO-*d_6_*) of complexes **1**–**4**.

Sn(IV) Complex	^13^C-NMR
**1**	168.3 (C=O), 156.7, 154.4, 143.1, 142.6, 141.3, 140.6, 137.0, 136.6, 136.4, 136.3, 135.2, 130.9, 129.8, 129.4, 128.6, 127.8, 126.8, 123.7, 122.7, 122.3, 119.1, 110.8, 109.7, 46.6 (CH_2_), 32.2 (Me), 29.2 (CH_2_), 21.2 (CH_2_), 16.9 (Me), 14.3 (Me)
**2**	171.0 (C=O), 156.6, 154.5, 143.2, 142.5, 137.0, 136.4, 135.2, 130.8, 129.6, 129.2, 128.7, 128.1, 127.8, 126.8, 123.7, 122.6, 119.1, 110.4, 109.8, 46.0 (CH_2_), 32.2 (Me), 29.2 (CH_2_), 28.4 (CH_2_), 28.2 (CH_2_), 26.7 (CH_2_), 21.2 (CH_2_), 16.9 (Me), 14.3 (Me), 14.1 (Me)
**3**	170.0 (C=O), 156.7, 154.4, 142.5, 141.5, 140.9, 140.7, 136.9, 136.4, 135.2, 132.7, 131.3, 130.8, 129.6, 129.2, 128.8, 127.7, 126.9, 123.7, 122.7, 122. 5, 119.0, 111.0, 109.9, 46.6 (CH_2_), 32.3 (Me), 29.2 (CH_2_), 21.2 (CH_2_), 16.9 (Me), 14.3 (Me)
**4**	169.6 (C=O), 156.6, 154.5, 143.2, 142.9, 141.0, 140.8, 137.1, 136.3, 135.2, 133.1, 130.1, 129.8, 129.4, 128.6, 127.8, 126.8, 126.8, 123.7, 122.7, 119.1, 110.8, 109.4, 46.6 (CH_2_), 32.2 (Me), 30.8 (CH_2_), 29.2 (CH_2_), 27.3 (CH_2_), 26.1 (CH_2_), 21.2 (CH_2_), 16.9 (Me), 14.3 (Me), 13.9 (Me)

**Table 5:** Porosity properties of complexes **1**–**4**.

Sn(IV) Complex	*S*_BET_ (m^2^·g^−1^) ^a^	*V*_total_ (cm^3^·g^−1^) ^b^	Pore Size (nm) ^c^
**1**	46.338	0.062	2.433
**2**	68.434	0.097	2.428
**3**	32.374	0.046	2.432
**4**	130.357	0.162	2.429

^a^ Brunauer–Emmett–Teller (BET) surface area; ^b^ pore volume was calculated at a relative pressure (*P*/*P^o^)* of 0.98 from the nitrogen adsorption isotherm; ^c^ Barrett–Joyner–Halenda (BJH) average pore diameter was calculated from the desorption data.

**Table 6:** Gas uptake capacities at 323 K and 50 bars of complexes **1**–**4**. ^a^

Sn(IV) Complex	CO_2_ Uptake (cm^3^·g^−1^)	CO_2_ Uptake (wt%)	H_2_ Uptake (cm^3^·g^−1^)	H_2_ Uptake (wt%)
**1**	18.2	3.6	1.1	0.009
**2**	20.5	4.0	0.7	0.006
**3**	16.5	3.3	0.5	0.006
**4**	35.0	7.1	1.1	0.013

^a^ Data were collected by the volumetric gas sorption method.

## Supplementary Materials

The following are available online.

## References

[1] Fanchi J.R., Fanchi C.J. (2016). Energy in the 21st Century.

[2] Leung D.Y.C., Caramanna G., Maroto-Valer M.M. (2014). An overview of current status of carbon dioxide capture and storage technologies. Renew. Sustain. Energy Rev..

[3] Ma S.Q., Zhou H.-C. (2010). Gas storage in porous metal–organic frameworks for clean energy applications. Chem. Commun..

[4] D’Alessandro D.M., Smit B., Long J.R. (2010). Carbon dioxide capture: Prospects for new materials. Angew. Chem. Int. Ed..

[5] Mastalerz M., Schneider M.W., Oppel I.M., Presly O. (2011). A salicylbisimine cage compound with high surface area and selective CO_2_/CH_4_ adsorption. Angew. Chem. Int. Ed..

[6] Ahmed D.S., El-Hiti G.A., Yousif E., Ali A.A., Hameed A.S. (2018). Design and synthesis of porous polymeric materials and their applications in gas capture and storage: A review. J. Polym. Res..

[7] Al-Mamoori A., Krishnamurthy A., Rownaghi A.A., Rezaei F. (2017). Carbon capture and utilization update. Energy Technol..

[8] Long J.R., Yaghi O.M. (2009). The pervasive chemistry of metal–organic frameworks. Chem. Soc. Rev..

[9] Eddaoudi M., Moler D.B., Li H., Chen B., Reineke T.M., O’Keeffe M., Yaghi O.M. (2001). Modular chemistry: Secondary building units as a basis for the design of highly porous and robust metal−organic carboxylate frameworks. Acc. Chem. Res..

[10] Tranchemontagne D.J., Mendoza-Cortés J.L., O’Keeffe M., Yaghi O.M. (2009). Secondary building units, nets and bonding in the chemistry of metal-organic frameworks. Chem. Soc. Rev..

[11] Johnson J. (2004). Putting a lid on carbon dioxide. Carbon sequestration, clean-coal research mark government response to climate-change threat. Chem. Eng. News.

[12] Yong Z., Mata V., Rodrigues A.E. (2002). Adsorption of carbon dioxide at high temperature—A review. Sep. Purif. Technol..

[13] Férey G. (2008). Hybrid porous solids: Past, present, future. Chem. Soc. Rev..

[14] Yan Y., Yang S., Blake A.J., Schroder M. (2014). Studies on metal–organic frameworks of Cu(II) with isophthalate linkers for hydrogen storage. Acc. Chem. Res..

[15] Zhao D., Timmons D.J., Yuan D., Zhou H.-C. (2011). Tuning the topology and functionality of metal−organic frameworks by ligand design. Acc. Chem. Res..

[16] Yaghi O.M., O’Keeffe M., Ockwig N.W., Chae H.K., Eddaoudi M., Kim J. (2003). Reticular synthesis and the design of new materials. Nature.

[17] Boot-Handford M.E., Abanades J.C., Anthony E.J., Blunt M.J., Brandani S., Mac Dowell N., Fernández J.R., Ferrari M.-C., Gross R., Hallett J.P. (2014). Carbon capture and storage update. Energy Environ. Sci..

[18] Kong Y., Shen X., Fan M., Yang M., Cui S. (2016). Dynamic capture of low-concentration CO_2_ on amine hybrid silsesquioxane aerogel. Chem. Eng. J..

[19] Builes S., López-Aranguren P., Fraile J., Vega L.F., Domingo C. (2015). Analysis of CO_2_ adsorption in amine-functionalized porous silicas by molecular simulations. Energy Fuels.

[20] Corvo M.C., Sardinha J., Casimiro T., Marin G., Seferin M., Einloft S., Menezes S.C., Dupont J., Cabrita E.J. (2015). A rational approach to CO_2_ capture by imidazolium ionic liquids: Tuning CO_2_ solubility by cation alkyl branching. ChemSusChem.

[21] Japip S., Wang H., Xiao Y., Chung T.S. (2014). Highly permeable zeolitic imidazolate framework (ZIF)-71 nano-particles enhanced polyimide membranes for gas separation. J. Membr. Sci..

[22] Huang Q., Eić M. (2013). Commercial adsorbents as benchmark materials for separation of carbon dioxide and nitrogen by vacuum swing adsorption process. Sep. Purif. Technol..

[23] Thomas A. (2010). Functional materials: From hard to soft porous frameworks. Angew. Chem. Int. Ed..

[24] Sumida K., Rogow D.L., Mason J.A., McDonald T.M., Bloch E.D., Herm Z.R., Bae T.-H., Long J.R. (2012). Carbon dioxide capture in metal–organic frameworks. Chem. Rev..

[25] Furukawa H., Ko N., Go Y.B., Aratani N., Choi S.B., Choi E., Yazaydin A.Ö., Snurr R.Q., O’Keeffe M., Kim J., Yaghi O.M. (2010). Ultrahigh porosity in metal-organic frameworks. Science.

[26] Banerjee R., Phan A., Wang B., Knobler C., Furukawa H., O’Keeffe M., Yaghi O.M. (2008). High-throughput synthesis of zeolitic imidazolate frameworks and application to CO_2_ capture. Science.

[27] Furukawa H., Yaghi O.M. (2009). Storage of hydrogen, methane, and carbon dioxide in highly porous covalent organic frameworks for clean energy applications. J. Am. Chem. Soc..

[28] Hossain M.M., de Lasa H.I. (2008). Chemical-looping combustion (CLC) for inherent CO_2_ separations—A review. Chem. Eng. Sci..

[29] Jones C.W. (2011). CO_2_ capture from dilute gases as a component of modern global carbon management. Annu. Rev. Chem. Biomol. Eng..

[30] Sanz-Pérez E.S., Murdock C.R., Didas S.A., Jones C.W. (2016). Direct capture of CO_2_ from ambient air. Chem. Rev..

[31] Ahmed D.S., El-Hiti G.A., Yousif E., Hameed A.S., Abdalla M. (2017). New eco-friendly phosphorus organic polymers as gas storage media. Polymers.

[32] Ghazi D., El-Hiti G.A., Yousif E., Ahmed D.S., Alotaibi M.H. (2018). The effect of ultraviolet irradiation on the physicochemical properties of poly(vinyl chloride) films containing organotin(IV) complexes as photostabilizers. Molecules.

[33] Ali M.M., El-Hiti G.A., Yousif E. (2016). Photostabilizing efficiency of poly(vinyl chloride) in the presence of organotin(IV) complexes as photostabilizers. Molecules.

[34] El-Hiti G.A., Alotaibi M.H., Ahmed A.A., Hamad B.A., Ahmed D.S., Ahmed A., Hashim H., Yousif E. (2019). The morphology and performance of poly(vinyl chloride) containing melamine Schiff bases against ultraviolet light. Molecules.

[35] Alotaibi M.H., El-Hiti G.A., Hashim H., Hameed A.S., Ahmed D.S., Yousif E. (2018). SEM analysis of the tunable honeycomb structure of irradiated poly(vinyl chloride) films doped with polyphosphate. Heliyon.

[36] Hashim H., El-Hiti G.A., Alotaibi M.H., Ahmed D.S., Yousif E. (2018). Fabrication of ordered honeycomb porous poly(vinyl chloride) thin film doped with a Schiff base and nickel(II) chloride. Heliyon.

[37] Yousif E., Ahmed D.S., El-Hiti G.A., Alotaibi M.H., Hashim H., Hameed A.S., Ahmed A. (2018). Fabrication of novel ball-like polystyrene films containing Schiff base microspheres as photostabilizers. Polymers.

[38] Balakit A.A., Smith K., El-Hiti G.A. (2018). Synthesis and characterization of a new photochromic alkylene sulfide derivative. J. Sulfur Chem..

[39] Altaee N., El-Hiti G.A., Fahdil A., Sudesh K., Yousif E. (2017). Screening and evaluation of poly(3-hydroxybutyrate) with *Rhodococcus equi* using different carbon sources. Arab. J. Sci. Eng..

[40] Altaee N., El-Hiti G.A., Fahdil A., Sudesh K., Yousif E. (2016). Biodegradation of different formulations of polyhydroxybutyrate films in soil. SpringerPlus.

[41] Yousif E., El-Hiti G.A., Haddad R., Balakit A.A. (2015). Photochemical stability and photostabilizing efficiency of poly(methyl methacrylate) based on 2-(6-methoxynaphthalen-2-yl)propanoate metal ion complexes. Polymers.

[42] Akram M.A., Nazir T., Taha N., Adil A., Sarfraz M., Nazir S.R. (2015). Designing, development and formulation of mouth disintegrating telmisartan tablet with extended release profile using response surface methodology. J. Bioequiv. Availab..

[43] Pejchal V., Holeček J., Nádvorník M., Lyčka A. (1995). ^13^C and ^119^Sn-NMR Spectra of some mono-*n*-butyltin(IV) compounds. Collect. Czech. Chem. Commun..

[44] Shahid K., Ali S., Shahzadi S., Badshah A., Khan K.M., Maharvi G.M. (2003). Organotin(IV) complexes of aniline derivatives. I. Synthesis, spectral and antibacterial studies of di- and triorganotin(IV) derivatives of 4-bromomaleanilic acid. Synth. React. Inorg. Met. Org. Chem..

[45] Rehman W., Baloch M.K., Badshah A., Ali S. (2005). Synthesis and characterization of biologically potent di-organotin(IV) complexes of mono-methyl glutarate. J. Chin. Chem. Soc..

[46] Thommes M., Kaneko K., Neimark A.V., Olivier J.P., Rodriguez-Reinoso F., Rouquerol J., Sing K.S.W. (2015). Physisorption of gases, with special reference to the evaluation of surface area and pore size distribution (IUPAC Technical Report). Pure Appl. Chem..

[47] Rabbani M.G., El-Kaderi H.M. (2011). Template-free synthesis of a highly porous benzimidazole-linked polymer for CO_2_ capture and H_2_ storage. Chem. Mater..

[48] Jin Y., Voss B.A., McCaffrey R., Baggett C.T., Noble R.D., Zhang W. (2012). Microwave-assisted syntheses of highly CO_2_-selective organic cage frameworks (OCFs). Chem. Sci..

[49] Yu H., Tian M., Shen C., Wang Z. (2013). Facile preparation of porous polybenzimidazole networks and adsorption behavior of CO_2_ gas, organic and water vapors. Polym. Chem..

[50] Katsoulidis A.P., Dyar S.M., Carmieli R., Malliakas C.D., Wasielewski M.R., Kanatzidis M.G. (2013). Copolymerization of terephthalaldehyde with pyrrole, indole and carbazole gives microporous POFs functionalized with unpaired electrons. J. Mater. Chem. A.

[51] Zhang W., Wojtas L., Aguila B., Jiang P., Ma S. (2016). Metal–metalloporphyrin framework modified with flexible *tert*-butyl groups for selective gas adsorption. ChemPlusChem.

[52] Meng L., Cheng Q., Kim C., Gao W.-Y., Wojtas L., Chen Y.-S., Zaworotko M.J., Zhang X.P., Ma S. (2012). Crystal engineering of a microporous, catalytically active fcu topology MOF using a custom-designed metalloporphyrin linker. Angew. Chem. Int. Ed..

