# Supplementary material for: Synthesis of Telmisartan Organotin(IV) Complexes and their use as Carbon Dioxide Capture Media
Source: Molecules. 2019 Apr 25;24(8):1631. doi: 10.3390/molecules24081631 (PMC6514663; doi:10.3390/molecules24081631)
Supplement: Supplementary file 1 [file molecules-24-01631-s001.pdf]

molecules

Article

Synthesis of Telmisartan Organotin(IV) Complexes and their use as Carbon Dioxide Capture Media

Angham G. Hadi [1], Khudheyer Jawad [1], Emad Yousif [2,*], Gamal A. El-Hiti [3,*], Mohammad Hayal Alotaibi [4], and Dina S. Ahmed [5]

[1] Department of Chemistry, College of Science, Babylon University, Babil 51002, Iraq; analhusainy@gmail.com (A.G.H.); khudheyer1965@gmail.com (K.J.)
[2] Department of Chemistry, College of Science, Al-Nahrain University, Baghdad 64021, Iraq
[3] Department of Optometry, College of Applied Medical Sciences, King Saud University, P.O. Box 10219, Riyadh 11433, Saudi Arabia
[4] National Center for Petrochemicals Technology, King Abdulaziz City for Science and Technology, P.O. Box 6086, Riyadh 11442, Saudi Arabia; mhhalotaibi@kacst.edu.sa
[5] Department of Medical Instrumentation Engineering, Al-Mansour University College, Baghdad 64021, Iraq; dinasaadi86@gmail.com
* Correspondence: emadayousif@gmail.com (E.Y); gelhiti@ksu.edu.sa (G.A.E.-H.); Tel.: +966-11469-3778 (G.A.E.-H.); Fax: +966-11469-3536 (G.A.E.-H.)

IR Spectra

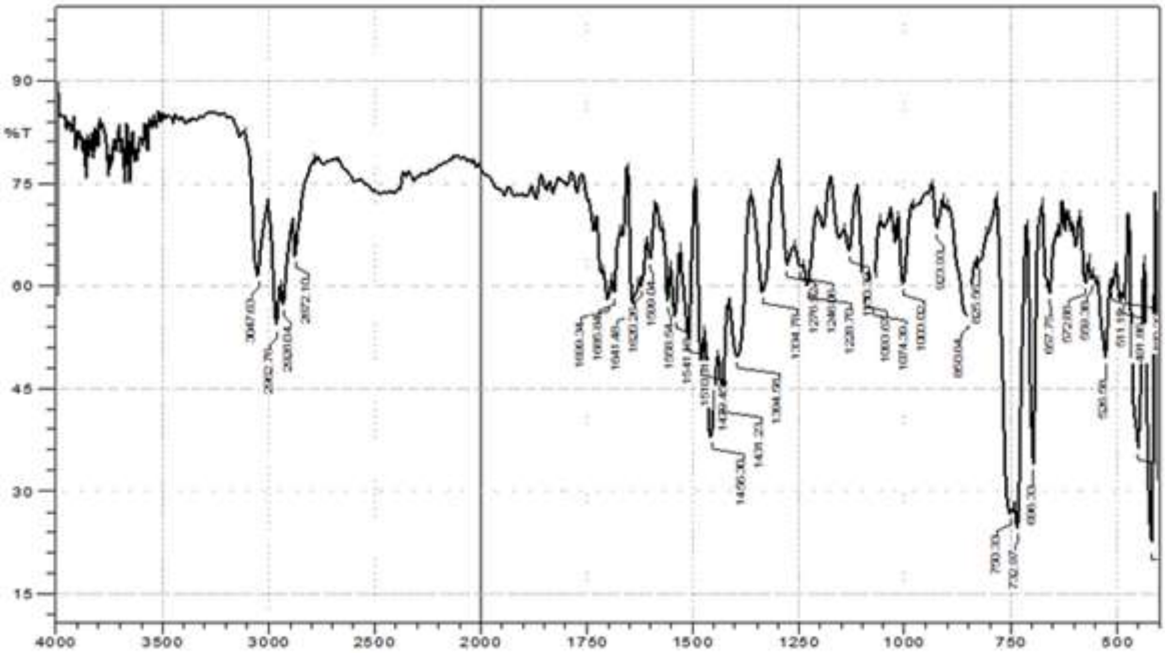

IR Spectrum of organotin(IV) complex **1**.

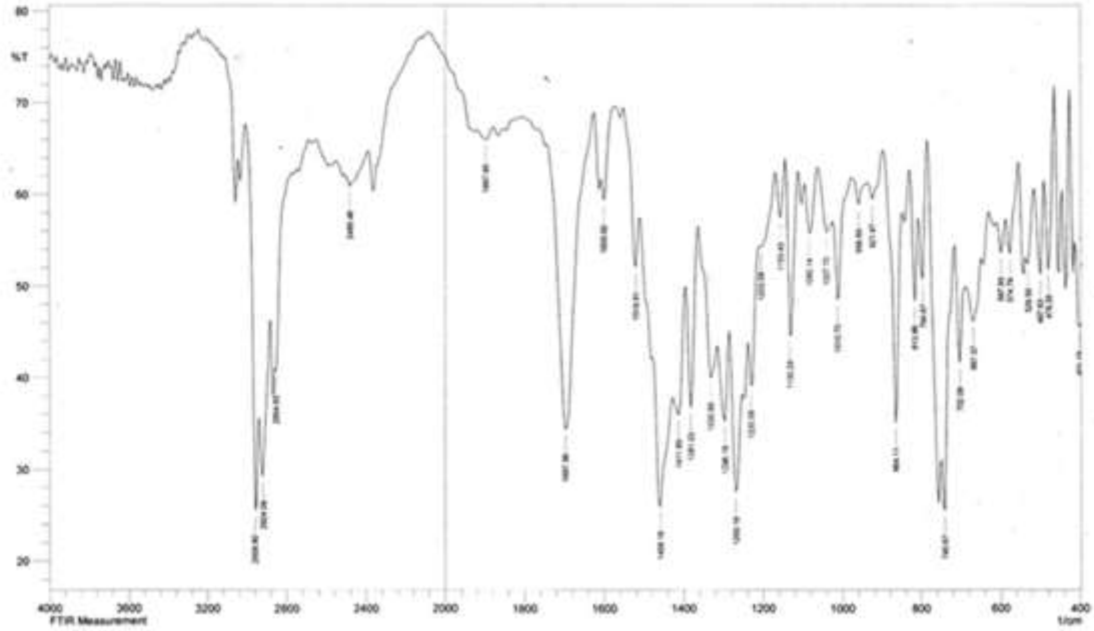

IR Spectrum of organotin(IV) complex **2**.

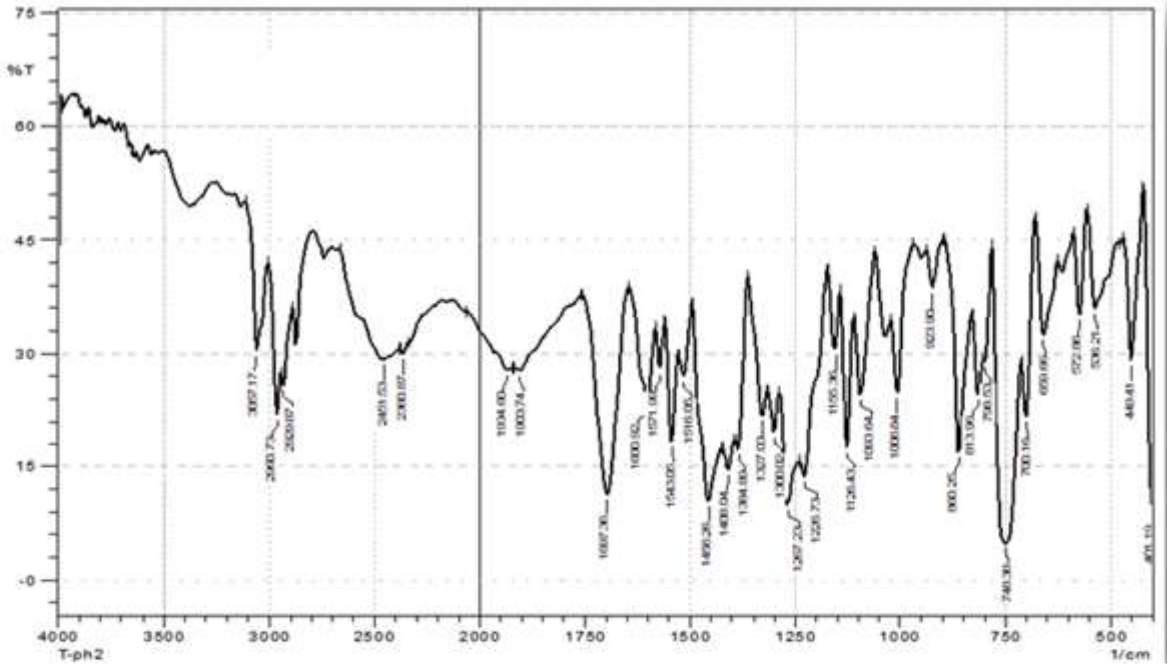

IR Spectrum of organotin(IV) complex **3**.

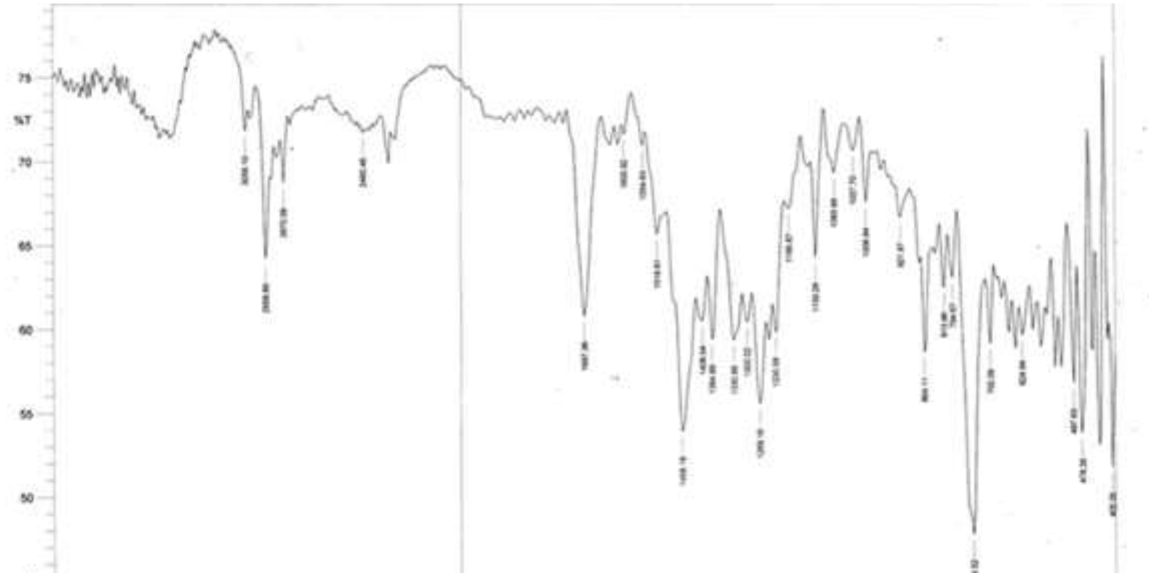

IR Spectrum of organotin(IV) complex **4**.

^{1}H NMR Spectra

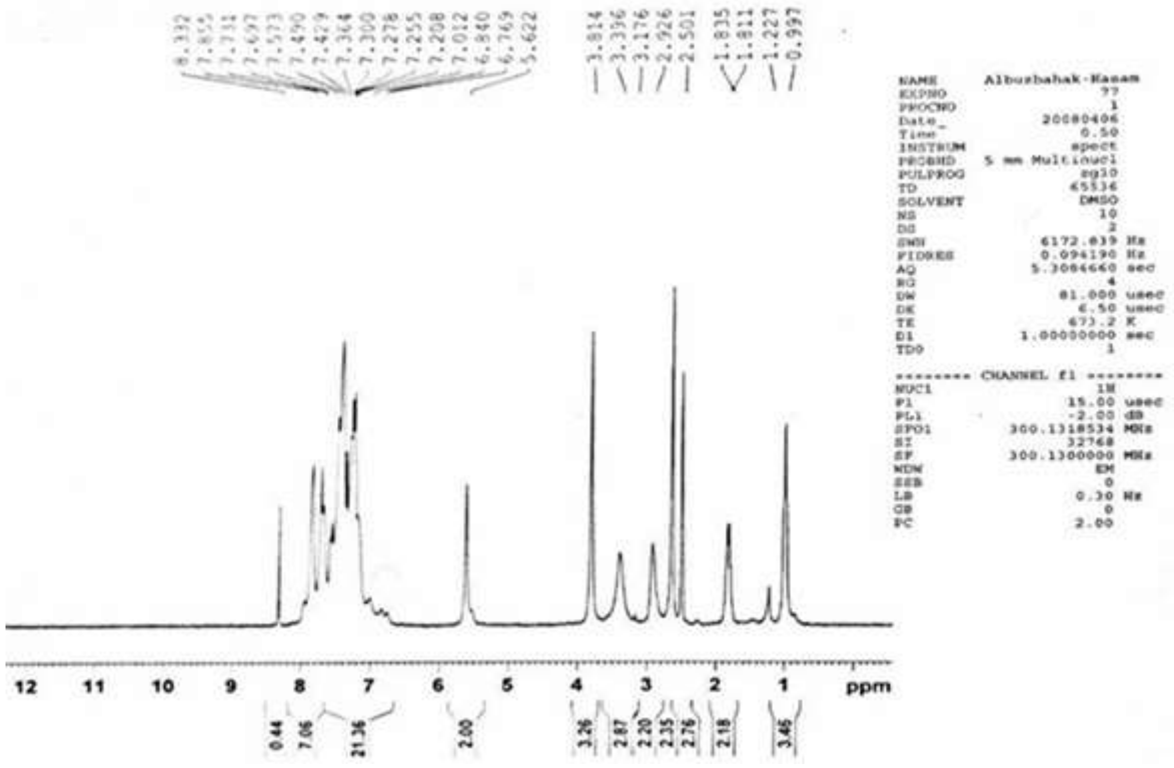

¹H NMR Spectrum of organotin(IV) complex **1**.

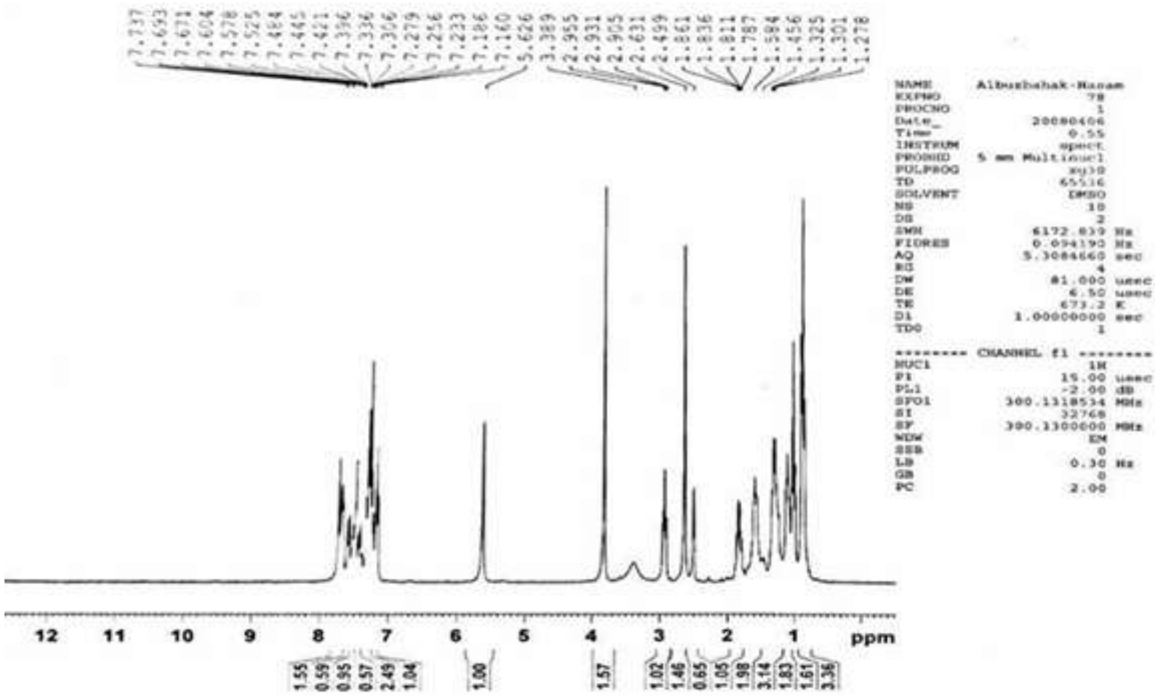

¹H NMR Spectrum of organotin(IV) complex **2**.

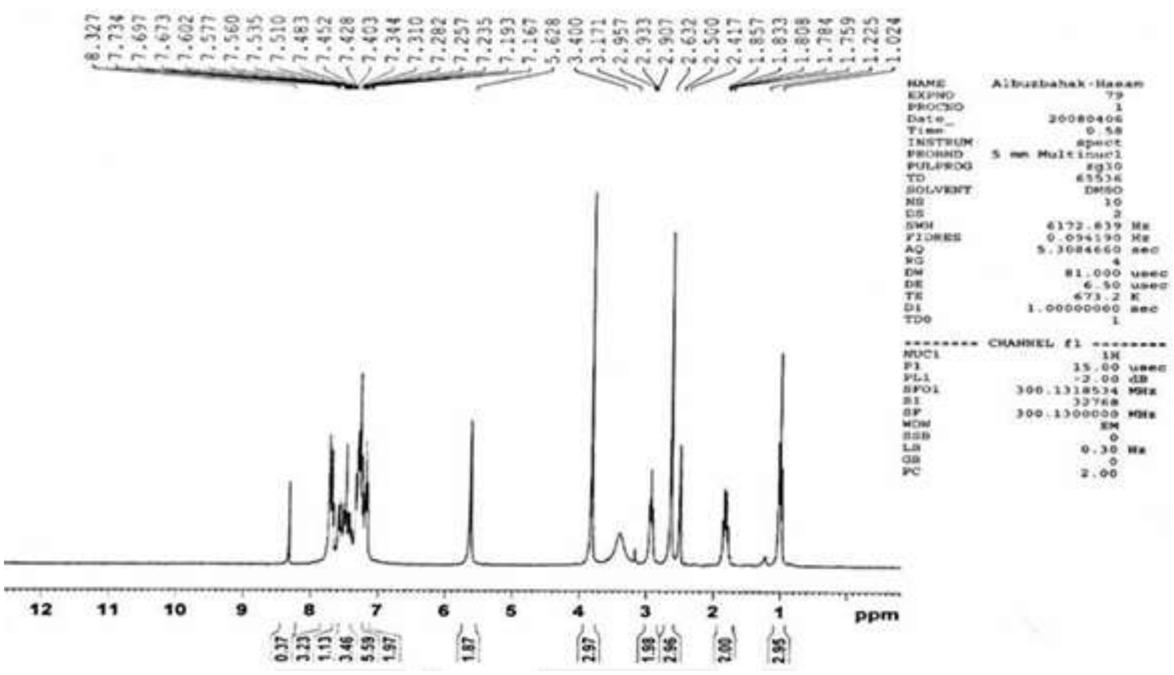

^{1}H NMR Spectrum of organotin(IV) complex **3**.

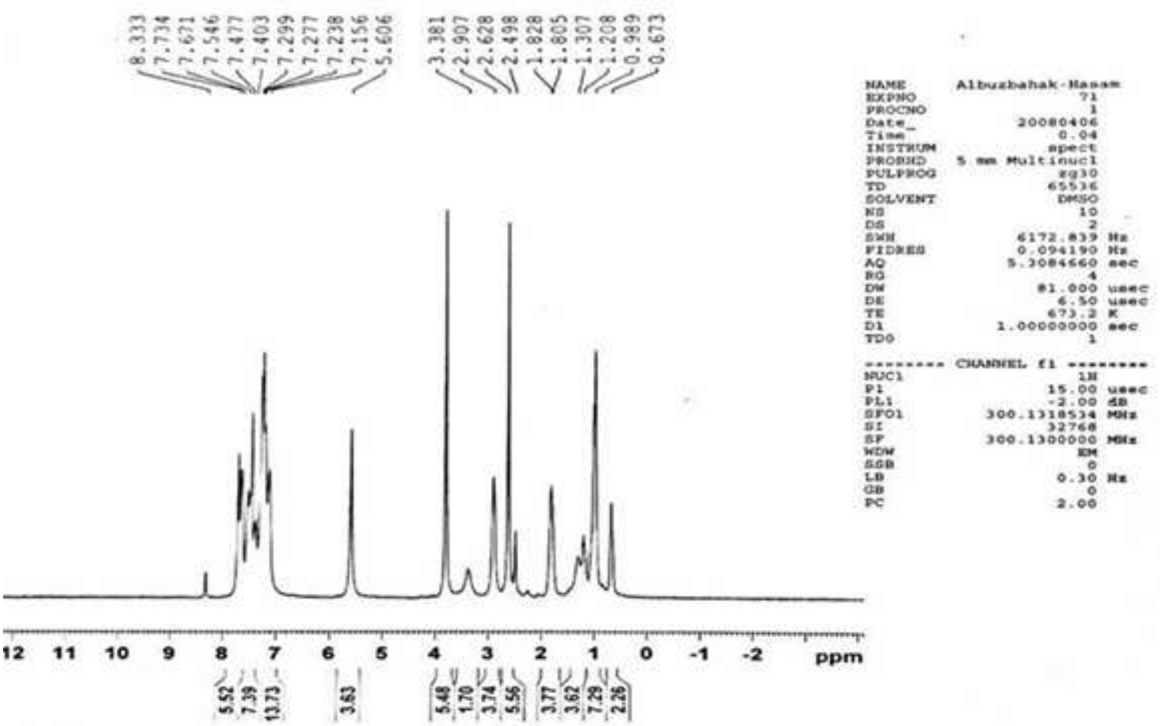

^{1}H NMR Spectrum of organotin(IV) complex **4**.

^{13}C NMR Spectra

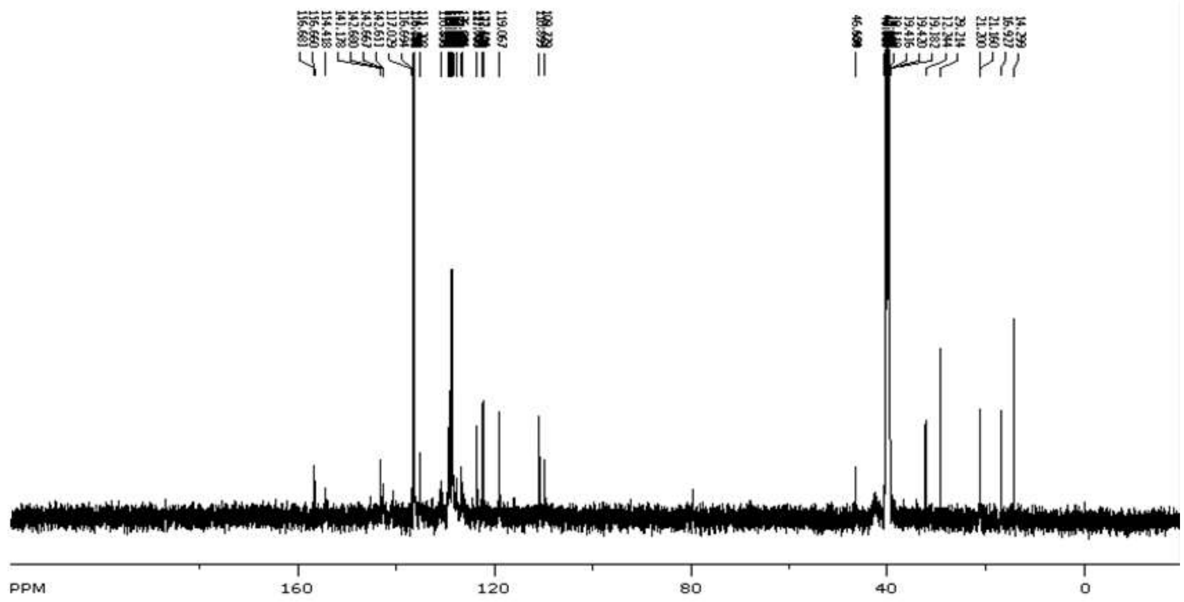

^{13}C NMR Spectrum of organotin(IV) complex **1**.

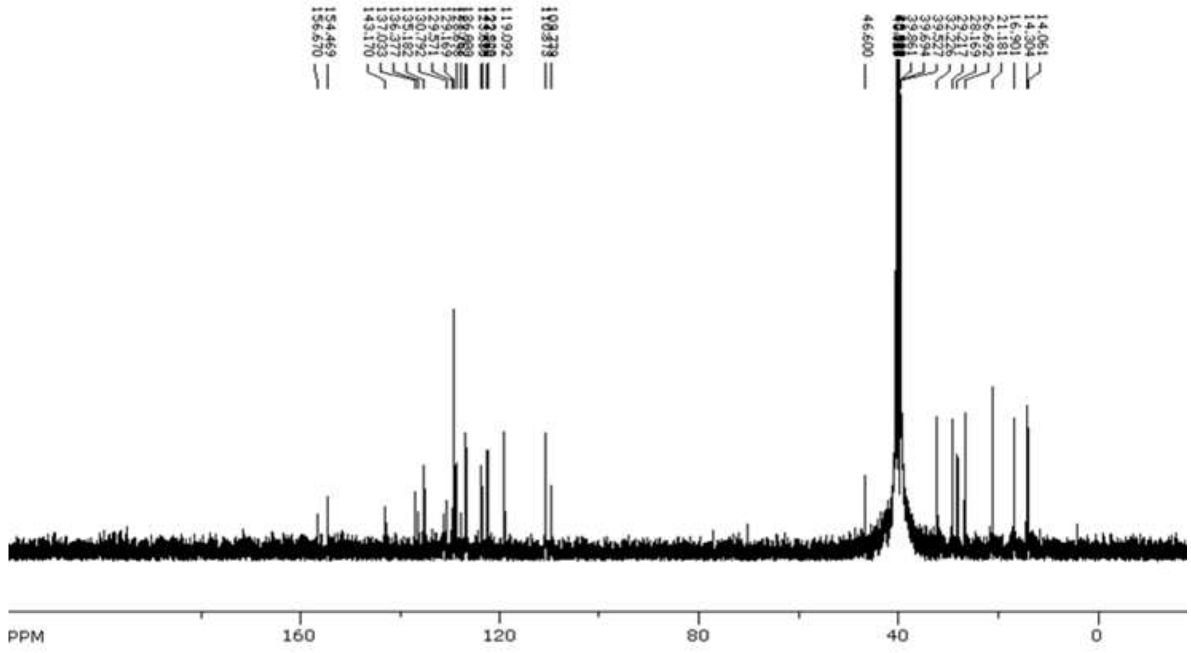

^{13}C NMR Spectrum of organotin(IV) complex **2**.

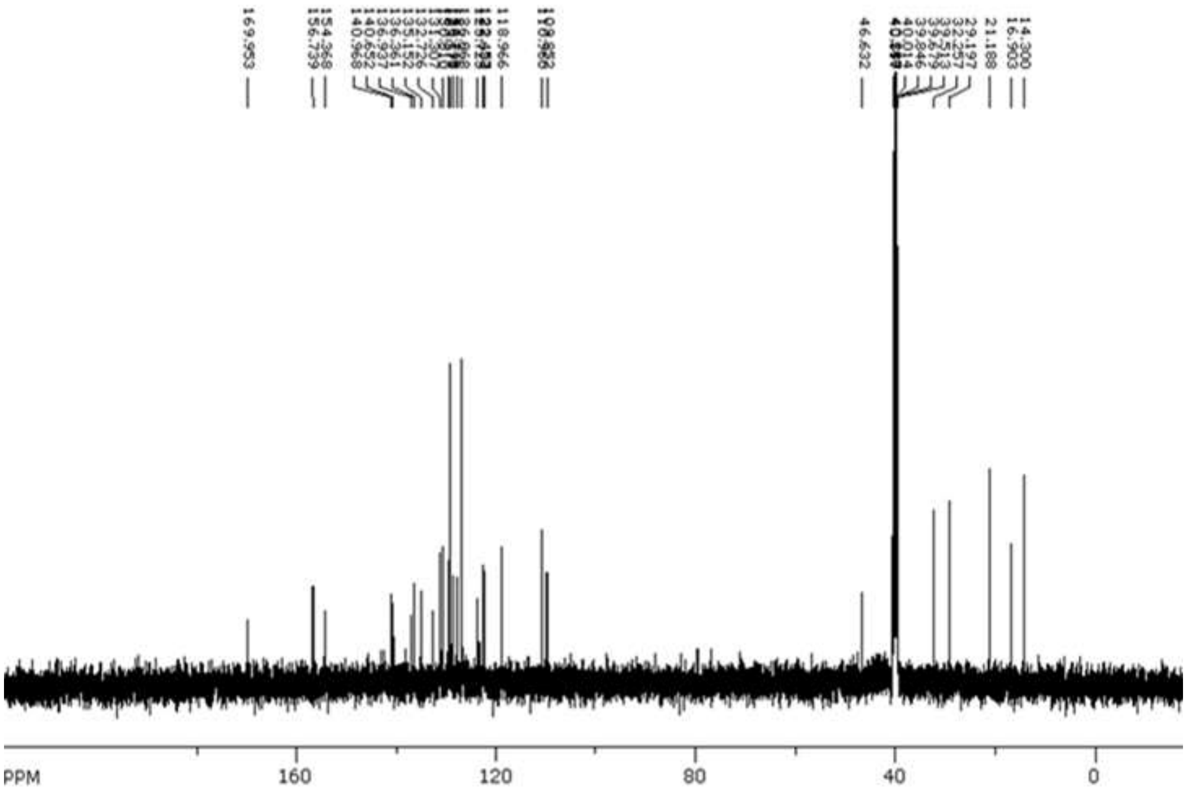

^{13}C NMR Spectrum of organotin(IV) complex **3**.

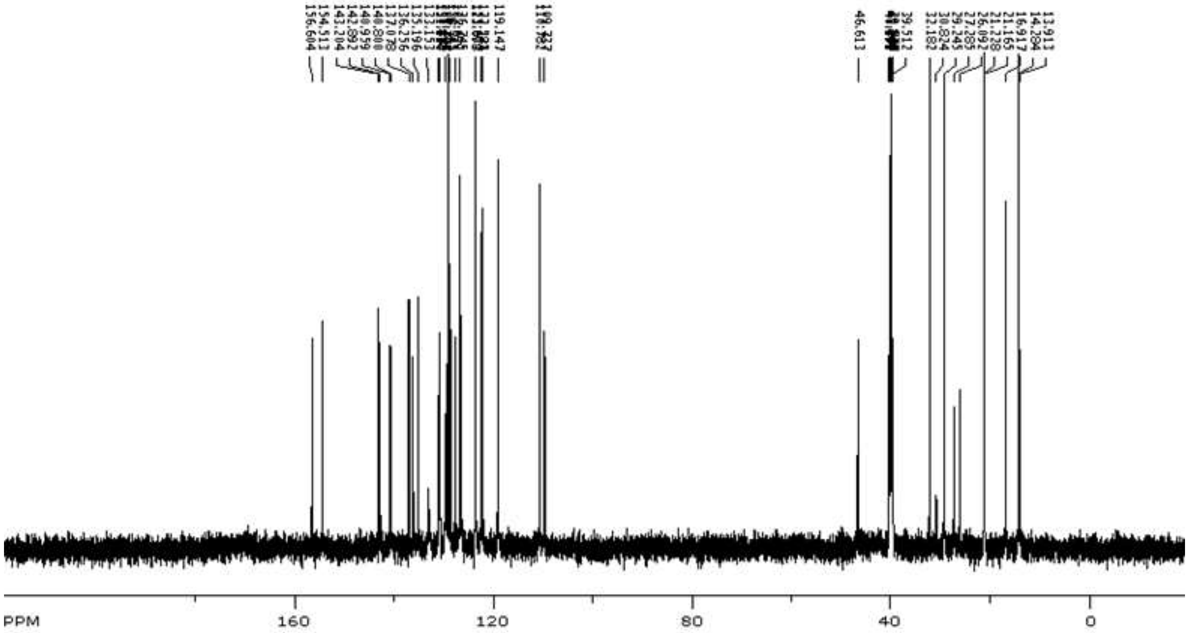

^{13}C NMR Spectrum of organotin(IV) complex **4**.

^{119}Sn NMR Spectra

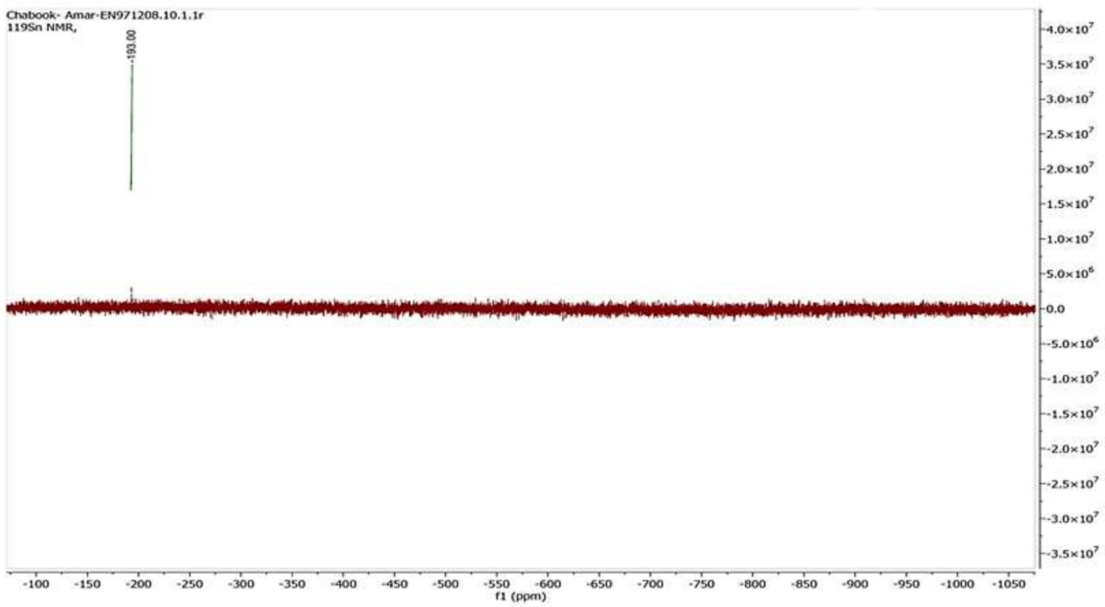

^{119}Sn NMR Spectrum of organotin(IV) complex **1**.

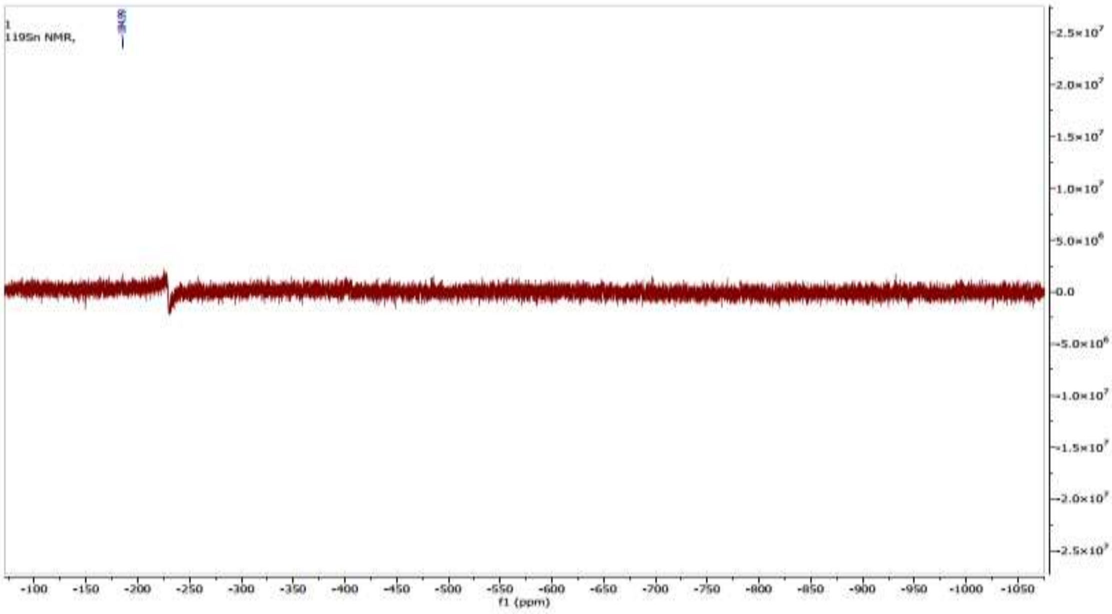

^{119}Sn NMR Spectrum of organotin(IV) complex **2**.

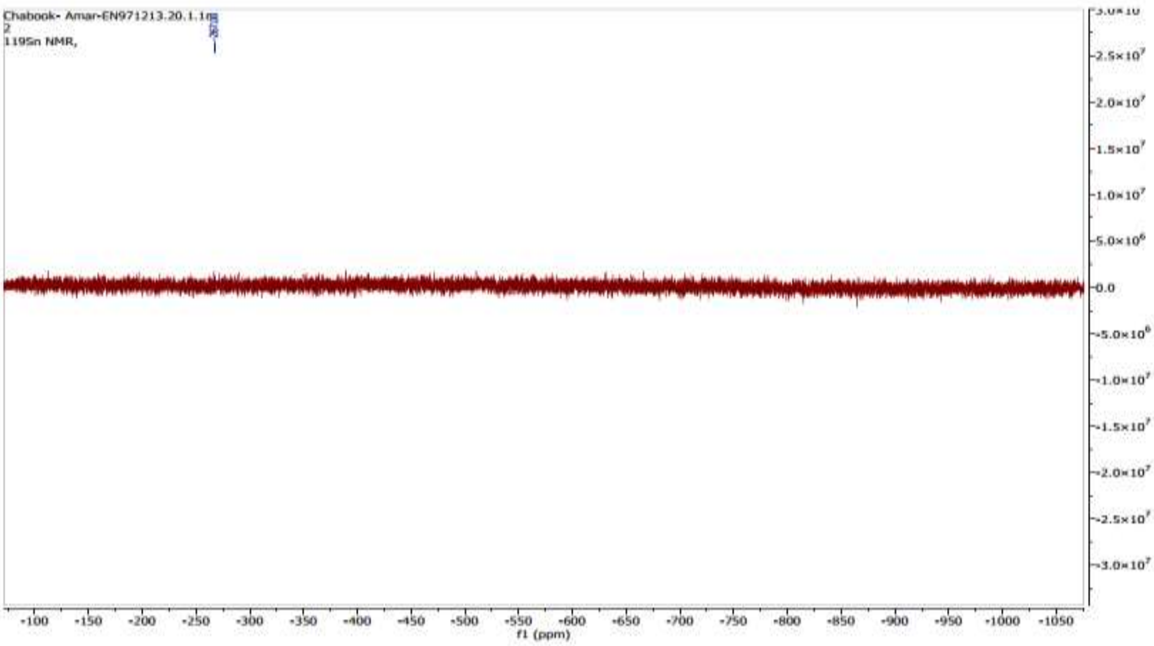

^{119}Sn NMR Spectrum of organotin(IV) complex **3**.

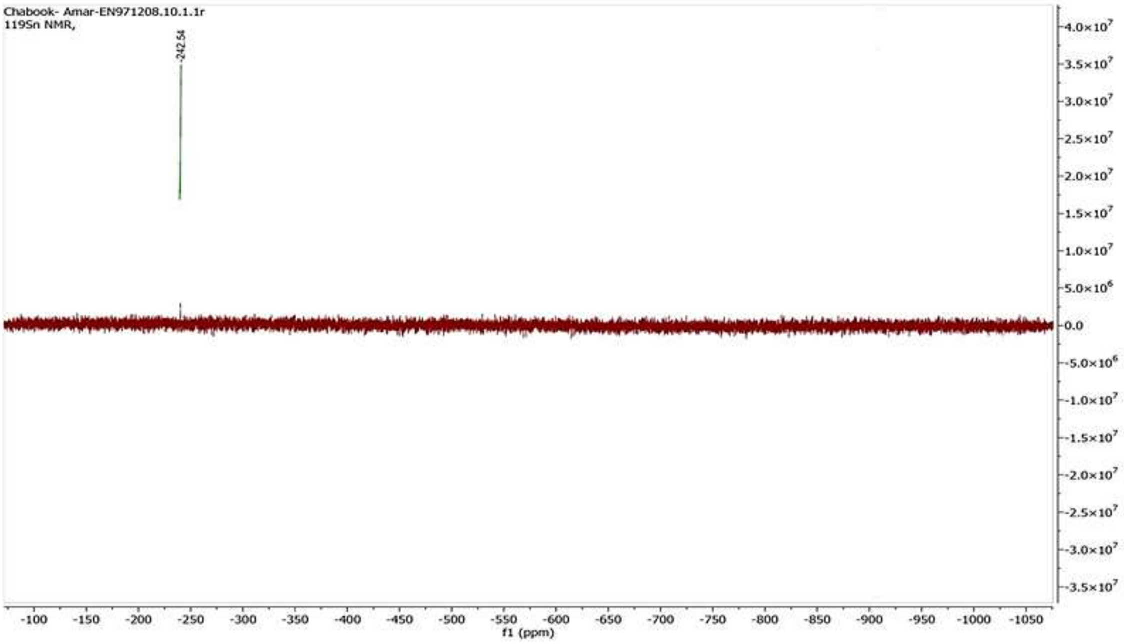

^{119}Sn NMR Spectrum of organotin(IV) complex **4**.
